# The Importance of Acromegaloid Physical Features for Clinical Practice

**DOI:** 10.1155/2023/5583344

**Published:** 2023-11-18

**Authors:** Ivona Perić, Gordana Zamolo, Boris Bezak, Jasna Klen, Dubravka Jurišić-Eržen

**Affiliations:** ^1^Department of Endocrinology and Diabetology, University Hospital Centre, Rijeka, Croatia; ^2^Department of Pathology, Faculty of Medicine, University Hospital Centre, University of Rijeka, Rijeka, Croatia; ^3^Department of Radiology, University Hospital Centre, Rijeka, Croatia; ^4^Faculty of Medicine, University of Ljubljana, Ljubljana 1000, Slovenia; ^5^Division of Surgery, Department of Abdominal Surgery, University Medical Centre Ljubljana, Ljubljana 1000, Slovenia; ^6^Department of Endocrinology, Faculty of Medicine, University of Rijeka, Rijeka, Croatia

## Abstract

Acromegaly and gigantism are hormonal disorders which develop as a consequence of chronic growth hormone hypersecretion. The prefix pseudo- is used to describe a certain clinical condition without a clearly proven characteristic of pathophysiological mechanism and basic biochemical features; pseudoacromegaly or acromegaloidism match the definition from above. In this case reports, we will try to provide a concise overview of diagnostic evaluation of acromegaloid physical appearance, while discussing two cases of patients who have similar clinical acromegaloid features as the first sign of the disease but have completely different etiologic backgrounds of their acromegalic appearance. The first case is of a 57-year-old male who presented with a marked acral growth and coarse facial features, but the diagnosis of secondary amyloidosis caused by multiple myeloma was confirmed just after biopsy of tongue and buccal mucosa. The second case is that of a 63-year-old male with an acromegaloid appearance caused by ectopic secretion of GH secreting lung carcinoma. The early diagnosis of ectopic acromegaly and pseudoacromegaly is still a challenging process. The key task is to confirm the GH axis abnormalities and establish the underlying disease, as a crucial step for faster treatment and need to avoid unnecessary therapeutic procedures to decreased mortality and improved quality of life.

## 1. Introduction

Patients with excess growth hormone usually show the following phenotype: typical physical characteristics with an emphasis on dominant prominent facial features with typical limb changes, above-average growth if the disease started at an early age and elevated levels of growth hormone and insulin growth factor (IGF-1). Hypersomatotropism is in most cases a primary pituitary disorder, while ectopic growth hormone (GH) secretion is exceptionally rare. Pseudoacromegaly or acromegaloidism is a clinical condition characterized by symptoms that clinically indicate an excess of growth hormone, but without proven abnormalities in the growth hormone/IGF-1 axis. A wide spectrum of clinical conditions, many of which are rare with overlapping clinical features or are genetically determined, can be the cause of pseudoacromegaly and differential diagnosis as such often represents a challenge. In this study, we aim to provide a comprehensive overview of two case reports of patients treated in Clinical Hospital Center, Rijeka, and show key differences between pseudoacromegaly and acromegaly caused by ectopic secretion of growth hormone.

## 2. Case Presentation

### 2.1. Case Report-Pseudoacromegaly

A 57-year-old male was referred to an endocrinologist from a hematologist to exude acromegaly because of the presence of marked acral growth and coarse facial features last year with no elements for any hematological disease. The first routine biochemical findings, done by a hematologist, exuded anemia, increased blood sedimentation, hypercalcemia, impaired renal function, alterations in the proteinogram, and protein excretion in 24-hour urine. The most important symptom was tongue enlargement accompanied with snoring, dysphagia, and speech and sleep difficulties. The patient also suffered from headaches in the occipital region and chronic pain in cervical and lumbar spine, with the pain extending to the right leg. His past medical history included essential hypertension, appendectomy at age of 11, perforation of stomach ulcer, and surgical treatment of discus hernia 1 year prior; while his family history was insignificant. At the time of this referral, a thorough clinical examination documented a height of 179 cm, weight of 99 kg, body mass index (BMI) of 30.9 kg/m^2^, and blood pressure (BP) of 115/70 mmHg. He had deep nasolabial folds, bulbous nose, blepharophimosis, drooping of eyelids, and enlarged frontal bones. The teeth were spread apart and he had a very deep voice. The most important symptom was macroglossia, which caused his reduced quality of life. The patient mentioned that, during last year, a slight enlargement of his hands and feet occurred with a weight loss of 20 kg, because of dysphagia. He denied loss of appetite. From other clinically significant findings, we documented hydrocele of left testis and referred the patient for evaluation to urology department where diagnosis of the tumor of urogenital tract was ruled out.

Routine laboratory tests including pituitary hormone levels were unremarkable and excluded diagnosis of diabetes and dyslipidemia. Endocrine evaluation showed a basal value of GH 5.13 mIU/L (*N* < 7.41 mIU/L) and IGF-1 7.9 nmol/L (N 6.6–24.3 nmol/L), and in oral glucose tolerance test (OGTT), GH levels was suppressed to 4.08 mIU/L at 60 minutes and 1.39 mIU/L at 120 minutes. Testosterone, calcitonin, parathyroid hormone, and cortisol levels were also within reference range, similar to calcium and magnesium levels and serum protein electrophoresis.

**Figure 1:**
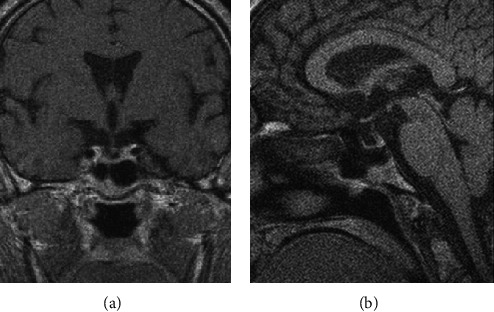
Bulky tongue reaching to the back surface of oropharynx causing obliteration of communication between nasopharynx and oropharynx (MRI of cervical region and cranium: (a) coronal view; (b) sagittal view).

Magnetic resonance imaging (MRI) of the cervical region and cranium was performed for evaluation and showed a voluminous bone and tongue reaching to the back surface of oropharynx without pathological post-contrast imbibition, consequently causing obliteration of communication between nasopharynx and oropharynx (Figure 1).

**Figure 2:**
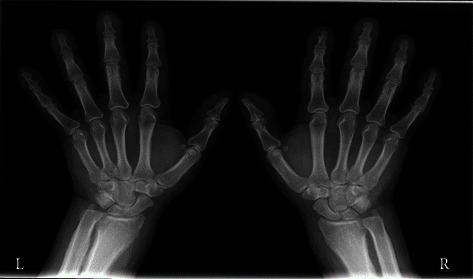
The radiogram of both hands.

**Figure 3:**
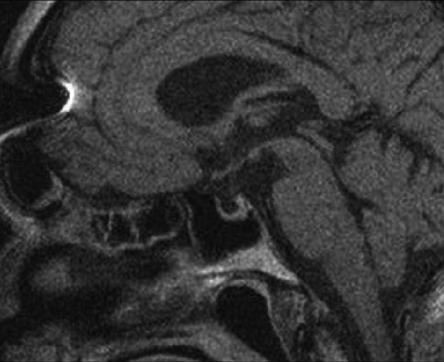
Sagittal MRI view of hypothalamic-pituitary area showed reduction of pituitary parenchyma.

A chest radiograph showed extensive pleural effusion and its cytology was described as a mixed pattern. From other significant image findings, a palmar radiograph showed a marked thickness of the periosteum, primarily of the proximal phalanges, with a marginal width of slightly more than 12 mm (Figure 2), and magnetic resonance imaging (MRI) of the hypothalamic-pituitary area showed reduction of pituitary parenchyma with incompetence of the diaphragm of sellae, more precisely partially empty sellae (Figure 3).

Somatostatin receptor scintigraphy detected minor focus of uptake at the upper abdominal region corresponding to pancreas, but low dose CT has not shown a visible substrate which could match mass expressing somatostatin receptors. On the other hand, the CT scan showed multiplied mesenterial lymph nodes.

**Figure 4:**
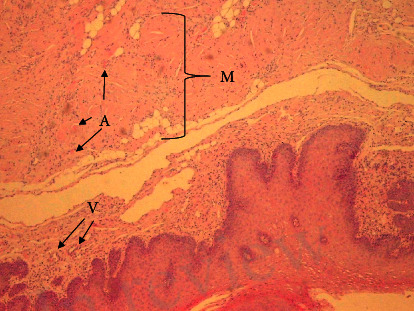
HE staining, magnification 4x: histologically, clusters of homogeneous eosinophilic material, also known as amyloid (A), can be seen in the mucosa, in the wall of smaller veins (V) and in the connective tissue between muscles (M).

**Figure 5:**
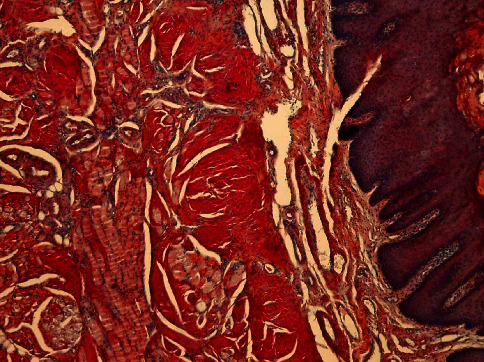
Congo red, 4x magnification; when dyeing with Congo red, the material is dyed bright red.

**Figure 6:**
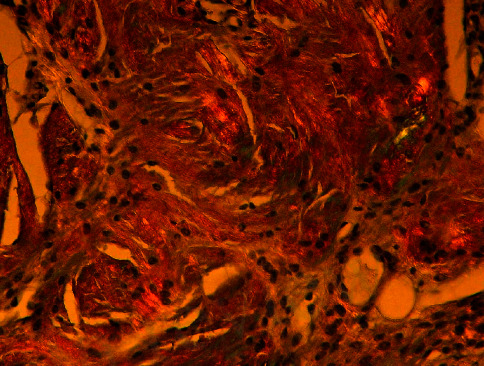
Congo staining, 20x magnification; under polarized light, the material gives positive birefringence and is greenish in color.

The next step in our diagnostic assessment was biopsy of tongue and buccal mucosa, which were affected organs. The pathohistological sample was subjected to green birefringence on polarization microscopy after Congo red staining, which corresponds to the diagnosis of amyloidosis (Figures 4–6). Depending upon the presentation and findings of amyloidosis, consultation was done with relevant specialists, hematologist, and further coordinated for multidisciplinary evaluation.

The hematological-biochemical analysis performed on the control showed a very small elevation of alpha-1-globulin 5.0 (normal 2.9–4.9%), decreased IgA 0.6 g/L (normal 0.7–4.0 g/L), and IgM 0.2 g/L (normal 0.4–2.3 g/L) in the proteinogram. Later, bone marrow aspiration and a bone marrow biopsy were performed; the diagnosis of multiple myeloma was confirmed and explained as the cause of secondary amyloidosis. In the process of undergoing hematological treatment, the patient died.

### 2.2. Case Report-Ectopic Secretion of Growth Hormone

A 63-year-old male was referred to an endocrinologist because of typical clinical symptoms of acromegaly. He complained of bilateral lower leg and knee pain for the duration of 1 year. In the mentioned period, he also noticed enlargement of his hands and feet with an increase in shoes size for 2 numbers. The patient denied headache and visual problems. A clinical examination revealed coarse face, deep creases on his forehead, broad nose, and bilateral conjunctivitis. His hands were symmetric and finger clubbing was noticeable, while feet were elongated and edematous. His past medical history included essential hypertension, diabetes mellitus, chronic obstructive pulmonary disease, chronic heart failure, and radiculopathy of lumbar region; while his family history was insignificant. At the time of this referral, a clinical examination documented a height of 175 cm, weight of 99 kg, BMI of 32.3 kg/m^2^, and BP of 130/90 mmHg with no other deviations.

Laboratory tests including pituitary hormone showed iron deficiency anemia (Hb 120 g/L, MCH 27 pg), leukocytosis, increased sedimentation rate but the value of GH was 13.13 mIU/L (*N* < 7.41 mIU/L), and IGF-1 25.4 nmol/L (N 6.6–24.3 nmol/L). The patient had mild diabetes for one year; so, in oral glucose tolerance test (OGTT), GH levels were 13.62 mIU/L at 60 minutes and 8.3 mIU/L at 120 minutes, respectively.

**Figure 7:**
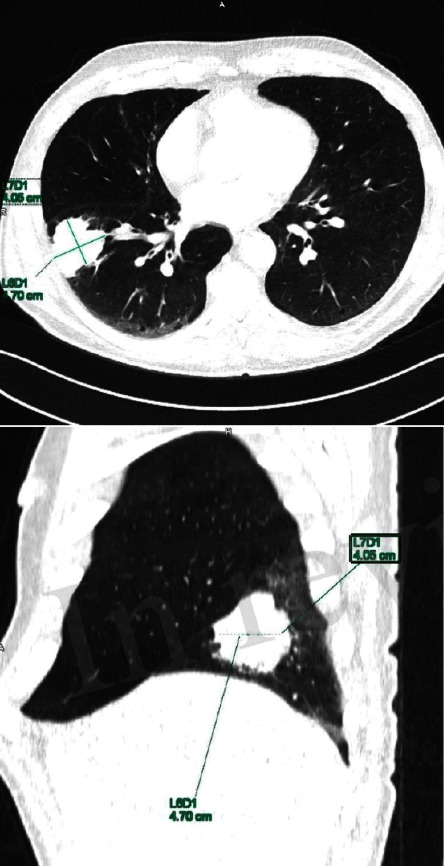
CT of thoracic organs showing an expansive formation with infiltration of the costal pleura in the lower right lung lobe accompanied with a nodular formation which seemed to most likely correspond to metastasis.

MRI of the hypothalamic-pituitary area showed no visible signs of microadenoma and palmar radiograph was unremarkable. During hospitalization, a routine chest radiograph and abdominal ultrasound were performed and showed nodule formation in the right lower pulmonary lobe accompanied by an inhomogeneous shadow in left lung and multiple hyperechogenic liver lesions. In addition to previous image findings, CT of thoracic and abdominal organs was performed. CT documented an expansive formation with infiltration of the costal pleura in the lower right lung lobe accompanied with a nodular formation which seemed to most likely correspond to metastasis. In the left upper lung lobe, an irregular areal was observed with potential malignant etiology. As an additional finding, multiple liver hemangiomas and nodular formations of the left and right adrenal glands, which seem to correspond to secondary lesions, were described (Figure 7).

Bronchoscopy biopsy confirmed pathology diagnosis of advanced squamous cell carcinoma and lung adenocarcinoma and the patient started with chemotherapy treatment as suggested by the oncologist.

## 3. Discussion

Acromegaly and gigantism are hormonal disorders that develop as a consequence of chronic GH hypersecretion, which in turn leads to excessive generation of IGF-1, the mediator of most of the effects of GH. Hypersomatotropism is in 99% of cases a GH-secreting adenoma in the pituitary gland, while ectopic secretion of GHRH and GH is a rare case [1]. Patients with excess of GH generally show three major attributes: characteristic physical appearance with prominent facial features and typical alterations in their extremities; tall stature, if the disease started before puberty; and elevated insulin-like growth factor-1 (IGF-1) levels and an elevated glucose-suppressed GH [2, 3].

Acromegalic physical appearance or tall stature is usually the reason why these individuals are being referred to endocrinologists. Except obvious clinical features and corresponding medical history, the assessment of GH secretion must be performed. Diagnostic approach includes measurement of basal fasting GH levels, measurement of GH after OGTT, IGF-1 measurement, and radiographic localization of pituitary adenoma [1]. Some of these patients could be healthy individuals with normal variants of tall growth and characteristic physical appearance, while others will have a confirmed case of acromegaly or pituitary gigantism, which are, in general, straightforward diagnoses upon assessment of the GH/IGF-1 axis [3]. Patients with acromegaly in more than 90% cases have basal fasting GH levels greater than 10 ng/mL, but single measurements are not reliable because of episodic secretion and other conditions such as acute illness, anxiety, cirrhosis, and diabetes mellitus type which can cause GH hypersecretion. IGF-1 is the mediator of most of the effects of GH; therefore, the screening should be performed by IGF-1 measurement according to current guidelines. Suppression after OGTT is still the simplest and most specific dynamic test for acromegaly. 60 minutes after oral admission of 100 g of glucose among healthy individuals, the GH level should reduce to less than 1 ng/mL, and if the patient has acromegaly, there is a lack of response; so, the GH level increases and shows no change or decreases but not to the value lower than 1 ng/mL. Almost all patients with acromegaly pituitary tumor can be identified by MRI, and among rare patients with normal neuroradiologic evaluation, an extra pituitary source of GH or GHRH is an option [1].

Ectopic acromegaly is an extremely rare disease, accounting for <1% of all cases of acromegaly, although one should be aware it exists. Signs, symptoms, and common hormonal evaluation do not differentiate clearly between tumors secreting GH and GHRH, and pituitary imaging does not provide proper diagnosis between pituitary adenoma and ectopic hypertrophy, and therefore could result in unnecessary surgery [4, 5]. Vast majority of GHRH-producing tumors are neuroendocrine tumors, which are quite often associated with MEN-1 syndrome and in our case, the source of ectopic secretion was neoplastic tissue of lung carcinoma.

However, some patients with physical features resembling acromegaly will have no abnormalities in the GH/IGF-1 axis. This scenario is termed pseudoacromegaly or acromegaloidism, as the prefix pseudo- is used to describe a certain clinical condition without a clearly proven characteristic pathophysiological mechanism and basic biochemical features. Its correct diagnosis can be challenging due to its rarity and wide spectrum of clinical conditions which can cause it, many of which are rare with overlapping clinical features or genetically determined [3, 6–9]. According to Mims, the term usage dates back to seventies and is defined as a “bodily condition resembling acromegaly but not due to a pituitary (or hypothalamic) disorder” [10]. In the mentioned report, it is described as a study which included 15 patients referred for evaluation of acromegaly, whose baseline and dynamic GH tests were negative. The patients' medical history included acromegalic faces and acral enlargement as well as prognathism, visceromegaly, hypertension, fatigue, headaches, arthralgias, paresthesia, oily odorous skin, hypertrichosis, and hyperpigmentation [3, 10]. Through the years, advanced molecular, genetic, and clinical studies have made the correct diagnosis possible. In 2018, Marques and associates published a study in which they provided a comprehensive overview of overlapping and rare conditions causing pseudoacromegaly while highlighting their similarities and differences with acromegaly and pituitary gigantism. Entities in review were arranged into 4 sections on the basis of acromegalic facial features with tall, normal, or short stature and tall stature without acromegalic features to aid physicians with the diagnosis of patients suffering pseudoacromegaly [3]. In our case report, pseudoacromegaly was caused by amyloidosis with multiple myeloma not recognized because of normal hematological findings. Amyloidosis can present with localized deposits or manifest as systemic disease involving multiple organs; in this case, amyloidosis led to macroglossia and consequential facial disfigurement.

Regarding to the literature and guidelines, there are no recommendations for the use of somatostatin analogs of the first and second generation in indication of multiple myeloma and lung cancer.

Acromegaly and pseudoacromegaly are heterogeneous clinical conditions whose presentation can be typical or atypical, depending on the etiology or the stage of disease progression. With the example of these two case presentations, we tried to demonstrate a modern diagnostic approach with the implementation of the latest guidelines. Also, by reviewing previous available literature, we have not encountered a significant number of case reports which described pseudoacromegaly caused by an underlying condition such as amyloidosis within multiple myeloma, and we believe it is important to report on the same in order to draw attention to this etiological possibility as well. On the other hand, we chose to report about the case of GH-secreting lung carcinoma to emphasize the fact that although rare, among individuals with an acromegaloid appearance ectopic secretion, it is always a possibility and an early diagnosis equates to decreased mortality and improved quality of life.

## 4. Conclusion

This report describes two patients who had similar clinical presentation but completely different etiologic background of their acromegalic appearance. As we have already mentioned earlier, the diagnosis of ectopic acromegaly and pseudoacromegaly is still a challenging process and the main task of the endocrinologist is to confirm or rule out GH axis abnormalities and to aid in establishing the underlying diagnosis, as a crucial step in planning treatment and avoiding unnecessary therapeutic procedures.

The crucial task is the early etiology detection of acromegalic appearance caused with or without the GH axis abnormalities, which will result in faster primary disease treatment with a goal in decreasing mortality and improving the quality of life. There is a need for emphasis in the guidelines for diagnosis and treatment of acromegaly with a highlight on protocol tools for excluding ectopic acromegaly and pseudoacromegaly.

## Data Availability

The data used to support the findings of this study are included within the article.
